# *Fusarium equiseti* as an Emerging Foliar Pathogen of Lettuce in Greece: Identification and Development of a Real-Time PCR for Quantification of Inoculum in Soil Samples

**DOI:** 10.3390/pathogens11111357

**Published:** 2022-11-15

**Authors:** George T. Tziros, Anastasios Samaras, George S. Karaoglanidis

**Affiliations:** Laboratory of Plant Pathology, Faculty of Agriculture, Forestry and Natural Environment, Aristotle University of Thessaloniki, P.O. Box 269, 54124 Thessaloniki, Greece

**Keywords:** *Lactuca sativa*, leaf spots, soilborne pathogens, qPCR, detection

## Abstract

Lettuce is the most commonly cultivated leafy vegetable in Greece, available in the market throughout the year. In this study, an emerging foliar disease observed in commercial farms has been associated to the pathogen *Fusarium equiseti*, a member of the *Fusarium incarnatum-equiseti* species complex (FIESC). Thirty *F. equiseti* isolates obtained from symptomatic lettuce plants were identified on the basis of morphology and evaluated for their pathogenicity. The isolates were further characterized using amplification and sequence analysis of the internal transcribed region (ITS-rDNA), and of the translation elongation factor 1-alpha (*TEF1-a*), calmodulin (*CAM*), beta-tubulin (*Bt*), and small subunit (*SSU*) genes. Moreover, a novel RT-qPCR assay was developed, designing a primer pair and a probe based on the *TEF1-a* sequences. This assay showed high specificity, amplifying *F. equiseti* DNA samples, while no amplification product was observed from samples of other common soilborne fungi. The generated RT-qPCR assay could be a useful tool for the detection and quantification of *F. equiseti* in soil samples deriving from fields cultivated with lettuce and other leafy vegetables, hosts of this specific pathogen.

## 1. Introduction

Lettuce is one of the most widely cultivated leafy vegetables in Greece, grown in the field or under protection in multitunnels or in greenhouses [1]. Lettuce cultivation in Greece covers an area of almost 3.200 hectares, in open fields and under greenhouses, and the production reached 46.643 tons [2]. A large number of soilborne and foliar pathogens affect lettuce cultivation in Europe [3]. Moreover, the intensive cultivation of leafy vegetables and the absence of adequate crop rotation, as well as climate change and the globalization of the seed market, constitute significant factors for the proliferation and subsequent spread of new pathogens [4]. Among these recently recorded pathogens, *Fusarium equiseti* could represent a significant plant pathogen for leafy vegetables, including lettuce and wild and cultivated rocket [3,5]. *F. equiseti* is a seedborne pathogen affecting many plant hosts [6]. The specific soil-inhabiting fungus survives as a saprotroph in soil or crop debris and thus this feature enables the ability of adapting easily to intensive cropping systems [3,4].

Lettuce is highly susceptible to *F. equiseti*, exhibiting severe yield losses at temperatures ranging from 25 to 35 °C and 1 to 3 h of high relative humidity [5]. The growers of leafy vegetables make use of chemical fungicides as most times the control of the environmental conditions is not practicable [5]. In addition, *F. equiseti* has the ability to produce a diversity of mycotoxins, thus constituting a potential risk for human and animal health [7,8].

*Fusarium equiseti* is a member of the *Fusarium incarnatum-equiseti* species complex (FIESC) which is comprised of species exhibiting significant genetic variability [9]. This species complex is divided into two clades, named *Incarnatum* and *Equiseti* [10,11]. FIESC includes more than 30 phylogenetically distinct species based on morphological identification and sequencing of more than one gene [7,12,13,14,15,16]. *F. equiseti* was defined as a distinct phylogenetic species within the subclade FIESC-14 [17]. 

For molecular identification, Maryani et al. (2019) used a multi-gene phylogeny that included partial fragments of the beta-tubulin (*Bt*), calmodulin (*CAM*), translation elongation factor 1-alpha (*TEF1-α*), the internal transcribed spacer region of the rDNA (ITS), the large subunit of the rDNA (*LSU*), as well as the RNA polymerase II large subunit (*RPB1*) and second-largest subunit (*RPB2*) genes. ITS, *TEF1-α*, *RPB1*, and *RPB2* were also used for the description of new FIESC species [14]. In addition, a species phylogeny inferred from partial nucleotide sequences from four housekeeping genes (*CAM*, *RPB2*, *TEF1-α*, and *Bt*) was carried out to identify FIESC species [7]. Furthermore, a multi-locus approach based on ITS, *TEF1-α*, *CAM*, *RPB1*, and *RPB2* was followed in order to distinguish species within the FIESC [15], while a three-gene (*TEF1*-α, *CAM*, and *RPB2*) phylogenetic inference was also used for the introduction of Latin binomials to unnamed FIESC phylo-species [16]. More recently, Matić et al. [12] identified isolates obtained from leafy vegetables using MLST analyses of four loci (*ΤEF1-α*, *CAM*, *Bt*, and IGS).

Pathogen identification based on morphological characteristics and microscopy is considered to be more or less subjective [18]. Molecular methods are highly sensitive and can detect and quantify microorganisms present even at low concentrations [19]. Real-time quantitative PCR (RT-qPCR) provides a reliable quantification of the population densities of several soilborne plant pathogens [20]. DNA can be extracted from various environmental samples, such as host tissues, soil, water, and air [20,21]. For instance, qPCR assays have already been developed for the detection and quantification of *Rhizoctonia solani* from soil and plant tissue samples derived from lettuce fields [22].

Accurate identification of *Fusarium* species is complicated and the morphological, biological, and especially phylogenetic approaches used most times lead to controversial results [23,24]. Thus, the development of cost-effective RT-qPCR assays for the detection and quantification of specific *Fusarium* spp. from soil samples could be of a great importance, especially for species infecting a broad range of plant hosts.

Foliar symptoms on lettuce consisting of circular to angular lesions which later become necrotic and cracked have been associated with anthracnose disease, caused by *Microdochium panattonianum* (syn. *Marssonina panattoniana*) [25] in Greece [26]. Interestingly enough, a recent review study on pathogens infecting leafy vegetables did not discuss anthracnose as an important disease of lettuce [3]. On the contrary, similar leaf spots caused by *F. equiseti* have already been recorded on lettuce in Italy [27].

Updated knowledge of the exact fungal species causing significant foliar symptoms and yield losses on lettuce cultivations in Greece is currently not available. Hence, the objectives of this research were to (a) associate the foliar symptoms observed on lettuce in the field with specific pathogens, (b) identify the isolates obtained from symptomatic plants using morphological characteristics and sequence analysis of the internal transcribed spacer ITS1-5.8S-ITS2 rDNA region (ITS-rDNA), translation elongation factor 1-alpha (*TEF1-a*), calmodulin (*CAM*), beta-tubulin (*Bt*), and small subunit (*SSU*) genes, (c) assess the pathogenicity of selected *F. equiseti* isolates on lettuce, and (d) develop an efficient RT-qPCR assay for the detection and quantification of the pathogen in soil samples collected from fields cultivated intensively with lettuce.

## 2. Materials and Methods

### 2.1. Pathogen Isolation

**Figure 1:**
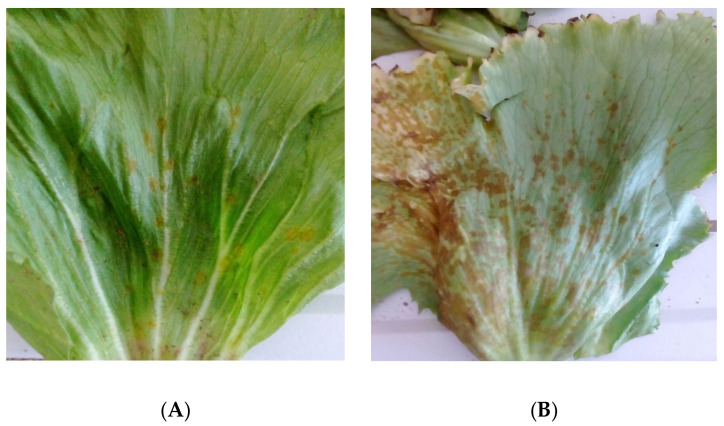
Leaf spot symptoms caused by *Fusarium equiseti* on *Lactuca sativa*. (**A**) Disease symptoms observed in the field, (**B**) severe symptoms developed on the outer leaves of lettuce plants collected from the same field.

A survey was conducted in the fields of a commercial farm specialized on the cultivation of leafy vegetables (Vezyroglou Farm, Alexandria, Imathia, Central Macedonia, Greece) in order to obtain isolates of the pathogen(s) causing foliar disease symptoms on Romaine lettuce plants. The symptoms recorded on the leaves and stems were small, 1 to 4 mm in diameter, dark yellow to brown, circular to angular leaf spots which later became necrotic, and which were sometimes cracked in the center (Figure 1). The disease was severe and widespread, and disease symptoms were observed on 20 to 35% of the leaves of almost all lettuce plants cultivated in an area of approximately 10 ha.

Diseased plants were arbitrarily selected at several sites in the fields and were transported to the laboratory in individual polyethylene bags to prevent cross-contamination. Tissues of symptomatic plants, taken from the margins of leaf and stems spots, were surface disinfested with a 10% sodium hypochlorite solution for 1 min and then were washed three times with sterilized distilled water. Tissue pieces were placed on potato dextrose agar (PDA; Oxoid, Thermo Fisher Scientific, Leiden, The Netherlands) amended with 1 mL of lactic acid 10% per 100 mL of PDA (pH 4.5), and incubated for 2 to 3 days, at 24 °C, in the dark. The yielded colonies were transferred onto new PDA dishes and from each petri dish, used for the initial isolation, only one isolate was kept in pure culture. The single-spore isolates obtained in this study were preserved at 4 °C for long-term storage.

### 2.2. Morphological Characterization

All isolates obtained were morphologically characterized following the protocols described by Leslie and Summerell [9]. Colony morphology and pigmentation were evaluated on PDA after 7 days of incubation at 24 °C in the dark. The isolates were evaluated for typical characteristics of FIESC species. Mycelial growth was estimated measuring the colony diameter in two perpendicular directions. Micromorphological characteristics of conidial shape and size were examined using water as mounting medium. Fungal structures were observed, captured, and measured (*n* = 100) using Zen 10 software of a Carl Zeiss, AXIO Lab A1 microscope.

### 2.3. Pathogenicity Assays

Lettuce seedlings of Romaine type (cv. Green Towers) were used for pathogenicity assays. Four-week-old lettuce seedlings were planted in plastic pots containing 200 g of sterilized substrate mixture (peat moss and perlite at a rate of 4:1). The candidate isolates were first cultured on PDA for 10 days to achieve sporulation. The conidia were then harvested and diluted to a final concentration of 1 × 10^6^ conidia/mL in sterile distilled water. The inoculum suspension was applied onto the above ground plant organs using an atomizer just before runoff.

Thereafter, the plants were covered with polyethylene bag for 3 days in a growth chamber to maintain high relative humidity. In the growth chamber the mean daily temperature was kept at 23 ± 2 °C. Twenty plants per isolate (three isolates were used in total) were arranged in a randomized block design and the experiment was repeated three times. Twenty plants sprayed with sterilized water were kept as controls. Ten days after inoculation, plants were examined for symptoms developed on their above-ground organs. Hence, in order to fulfill Koch’s postulates, isolations were carried out from non-inoculated plants and from the ones inoculated with the evaluated isolates. Additionally, the isolates derived from artificially inoculated plants were amplified with *TEF1-α* genes and the amplicons were sequenced.

### 2.4. DNA Extraction

Petri dishes (diameter 9 cm) containing *ca*. 20 mL PDA, overlain with sterilized cellophane sheets (gel drying frames, Sigma–Aldrich Chemie GmbH, Taufkirchen, Germany), were inoculated with mycelial plugs (4 mm in diameter) and incubated for 3–5 days in the dark at 24 °C. The mycelium was scraped from each plate, lyophilized, and ground to a fine powder. Genomic DNA was extracted from this material using the DNeasy Plant Mini Kit (Qiagen, Hilden, Germany) following the manufacturer’s instructions. The concentration of the extracted DNA was measured using a P330 nanophotometer (Implen GmbH, Munich, Germany).

### 2.5. PCR Amplification, Sequencing, and Phylogenetic Analysis

Initially, the ITS1-5.8S-ITS2 region was amplified with primers ITS1/ITS4 [28]. The translation elongation factor 1-alpha (*TEF-1α*) gene was amplified with primers EF-1/EF-2 [29], while partial amplification of calmodulin (*CAM*), and beta-tubulin (*Bt*) was performed with CL1/CL2A [30] and Bt2a/Bt2b [31] primers, respectively. In addition, partial amplification of small subunit (*SSU*) was performed with the specific primers PNS1/NS41 [32]. Aliquots of the PCR products were loaded on 1.0% agarose gel in Tris-acetate-EDTA buffer with Midori Green Advance gel stain (Nippon, Duren, Germany). PCR products were purified with PureLink PCR Purification Kit (Invitrogen, Carlsbad, CA, USA) and custom sequenced (CEMIA).

All the sequences generated in this study were initially visualized by ChromasLite (Technelysium, South Brisbane, Australia), then visually aligned and two representative sequences were deposited in the GenBank. The sequence data obtained were compared by BLAST search on the National Center for Biotechnology Information (NCBI) database to determine the species, searching for similarities between the sequences obtained in this study and already existing sequences in the database.

In accordance with the primer/probe design which is described in Section 2.6.1, the best hits obtained from the blast searches were downloaded and used in the construction of a phylogenetic tree. Phylogenetic analysis was conducted using MEGA version 7.0, and maximum likelihood (ML) method was used to generate the phylogenetic tree from the *TEF1-α* gene. Bootstrap values were obtained from 1000 replicates and distances were calculated using Kimura-2p in both phylogenetic inferences. The sequence of *Didymella pinodella* (GenBank accession no. MK525067.1) was used as an outgroup for rooting the phylogenetic tree.

### 2.6. Development of a Fusarium equiseti-Specific qPCR Assay

#### 2.6.1. RT-qPCR Primers/Probe Design and Specificity

Partial gene sequences of the translation elongation factor 1-alpha (*TEF1-α*) gene were evaluated for the presence of suitable regions to design primers and a probe specific for *F. equiseti*. *TEF1-α* sequences, corresponding to *F. equiseti*, the *Equiseti* clade of the FIESC, and to other *Fusarium* spp., were retrieved from the GenBank database and examined for regions potentially unique to *F. equiseti*.

**Figure 2:**
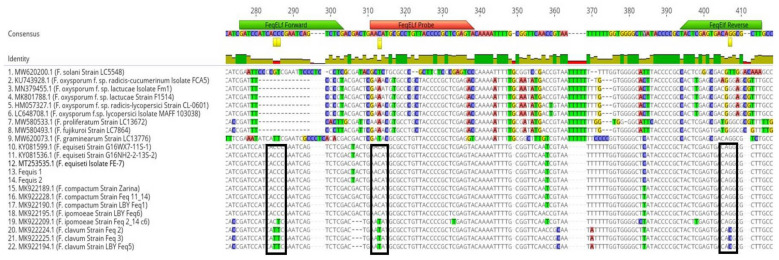
Nucleotide alignment of partial genomic sequences of two representative isolates obtained in this study (Fequis 1 and 2), based on the amplification product of the translation elongation factor (*TEF1-a*) gene. Sequences of *Fusarium equiseti*, *Equiseti* clade, and *Fusarium* spp. isolates retrieved from NCBI database were also included in the alignment. *TEF1-a* locations of primers (green arrows) and probe (red arrow) designed for the RT-qPCR assay developed in this study are indicated. Black frames in the figure indicate the nucleotides presented only in *Fusarium equiseti* isolates, in the regions of designed primers and probe.

The nucleotide alignment of *F. equiseti TEF1-α* sequences obtained in this study and retrieved from the NCBI database was used to design the RT-qPCR primers-probe set (Figure 2). The sequences were aligned using the MAFT alignment tool available in Geneious V9.1.8. Based on the alignment, a set of two primers (FeqELf-F and FeqELf-R) and a probe (FeqELf-Pro) were designed to cover all the variability detected in the selected area, amplifying a PCR product of 128 bp (Table 1). The TaqMan probe sequence was labeled with the fluorescent reporter dye 6-FAM (6-fluorescein amidate) and the Iowa Black Fluorescent Quencher (IABkFQ) at the 5′ and 3′ ends, respectively, and an internal ZEN quencher. For primer and probe selection, specific criteria were followed as described by Šišić et al. [18]. Potential secondary structures, such as hairpin, self-dimer, and hetero-dimer interactions were checked using the oligoanalyzer tool from Integrated DNA Technologies (https://www.idtdna.com/pages/tools/oligoanalyzer (accessed on 15 February 2022)). The specificity of the primers and probe was scanned against the NCBI database using basic local alignment search tools.

The designed primers were subjected to conventional PCR to confirm their specificity. 10 μL of 5×One Taq Standard Reaction Buffer (New England BioLabs Inc., Hitchin, UK), 1.0 μL of dNTPs at 10 μM, 1.0 μL of each primer at 0.5 μM (IDT, Leuven, Belgium), 0.25 μL of Taq DNA polymerase (New England BioLabs Inc., Hitchin, UK), 5 mL of DNA template, and 31.75 mL of sterile distilled water were added to each reaction tube to make a final volume of 50 μL. PCR was performed with a thermocycler (SensoQuest, Labcycler, Germany) with the following amplification conditions: initial denaturation step at 94 °C for 5 min; 30 cycles of denaturation at 94 °C for 30 s, primer annealing at 55 °C for 30 s, and extension at 72 °C for 1 min and a final extension at 72 °C for 5 min. The PCR step also included DNA samples from seven common soilborne fungal pathogens, as well as no-template samples as negative controls.

The primer/probe set was validated for specificity using a fungal species panel by RT-qPCR amplification following the protocol described below in Section 2.6.2. The validation panel included DNA extracts from a range of fungal and Oomycete species, including *F. equiseti* isolates obtained in this study, *Rhizoctonia solani* and *Pythium ultimum* commonly occurring in lettuce cultivated fields [1], *F. oxysporum* f.sp. *lactucae* from lettuce plants showing wilt symptoms, as well as other *Fusarium* spp. (*F. proliferatum*, *F. fujikuroi*, *F. gramineraum*, *F. solani*, *F. oxysporum* f.sp. *radicis-lycopersici*, and *F. oxysporum* f.sp. *radicis-cucumerinum*) available at the fungal collection of Plant Pathology Lab, AUTh. In addition, DNA extracted with the DNeasy Power Soil kit (Qiagen, Hilden, Germany), from soil samples collected from the surveyed field was also included in the assay.

#### 2.6.2. qPCR Conditions of *Fusarium equiseti* Assay

DNA extracted from single-spore cultures was amplified with the primer/probe set developed in this study. The reaction mixture for each DNA sample, consisted of 3 μL of DNA template, 10 μL of Luna Universal Probe qPCR Master Mix (New England Biolabs, Ipswich, MA, USA), 500 nM of each of the forward and reverse primers, 250 nM of the probe, and 0.1 μL BSA (New England Biolabs, Ipswich, MA, USA) in a final volume of 20 μL. For each sample triplicate reactions were performed and molecular grade water (Panreac, AppliChem, Barcelona, Spain) was used as negative control. The RT-qPCR reactions were performed using a Strategene Mx3005P qPCR System (Agilent Technologies, Santa Clara, CA, USA) on 96-well plates. Amplification conditions consisted of a single cycle at 94 °C for 1 min, followed by 40 cycles of 94 °C for 30 s, 55 °C for 30 s, and 94 °C for 10 s. Data were collected in the last holding stage of each cycle and the results were analyzed using the Strategene MxPro-Mx3005P Software (Version 4.00; Agilent Technologies, Santa Clara, CA, USA).

#### 2.6.3. Preparation of RT-qPCR Standard Curve

For the preparation of the standard curve, the specific fragment targeted by the qPCR was amplified by conventional PCR using primers FeqELf-F and FeqELf-R. The 128-bp PCR product was purified using PureLink Purification Kit (Invitrogen, Carlsbad, CA, USA), and then inserted in the pUC19 vector contained in the NEB PCR Cloning kit (New England Biolabs, Ipswich, MA, USA) following the manufacturer’s instructions, and cloned into 10-beta Competent *Escherichia coli* cells contained in the same kit. Transformant colonies were selected by ampicillin resistance. Plasmid DNA purification was carried using the Monarch Plasmid Miniprep Kit (New England Biolabs, Ipswich, MA, USA) following the manufacturer’s instructions and the concentration of the extracted DNA was measured using a P330 nanophotometer (Implen GmbH, Munich, Germany).

The Avogadro constant (6.023 × 10^23^ molecules/mol) was used to estimate the number of plasmid molecules. Three replicates of ten-fold serial dilutions (10^9^ down to 10^1^ plasmid copies/μL) were prepared and used to generate the standard curve. 1 μL from each dilution was used in RT-qPCR in order to determine the amplification efficiency, the dynamic range of quantification, and the detection limit of the assay. The threshold cycle (Ct) values were automatically calculated with the Strategene MxPro-Mx3005P Software (Version 4.00; Agilent Technologies, Santa Clara, CA, USA) and the standard curve was created by plotting the Ct values versus the logarithm of the concentration of each serial dilution. For each serial dilution, the mean Ct value was estimated, and the number of DNA molecules in each sample was calculated by incorporation of the mean Ct value to the standard curve equation.

### 2.7. Validation of the Assay in Artificially Inoculated Substrate with F. equiseti Spores

One selected isolate (Fequis 1) identified in this study was used to inoculate a sterile substrate (peat moss, Hawita Professional), autoclaved at 121 °C for 60 min twice, at an interval of 24 h. The pathogen inoculum was prepared as described above in Section 2.3 of this manuscript. One mL of the *F. equiseti* conidial suspensions (10^2^, 10^3^, 10^4^, 10^5^, 10^6^ conidia mL^−1^) were added to the sterile substrate, mixed meticulously, and then placed into 50 mL Falcon tubes. Controls consisting of sterile substrate were inoculated solely with sterile distilled water. The substrate-*F.equiseti* conidia mixture was subsequently dried at 60 °C for 24 h. For each concentration, DNA extraction was performed in triplicate using the DNeasy Power Soil kit and amplified using the RT-qPCR assay developed in this study.

## 3. Results

### 3.1. Pathogen Identification

**Figure 3:**
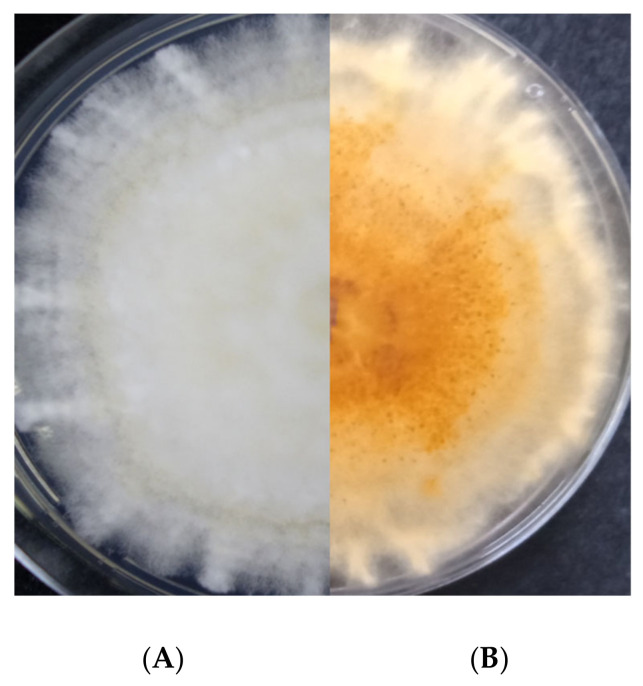
Colony appearance of *Fusarium equiseti* on PDA after 7 days of incubation at 24 °C in the dark. (**A**) dorsal and (**B**) ventral view.

Morphological characteristics tally with those described before for FIESC [9,15,16]. The isolates gave rise to white mycelium which later produced a pale brown to dark brown pigmentation (Figure 3). The average growth rate varied from 1.9 to 3.6 mm/day. Microconidia were aseptate, hyaline, ovoid, fusiform (ellipsoidal), or slightly curved, and 8.2–13.8 × 2.0–4.5 μm.

**Figure 4:**
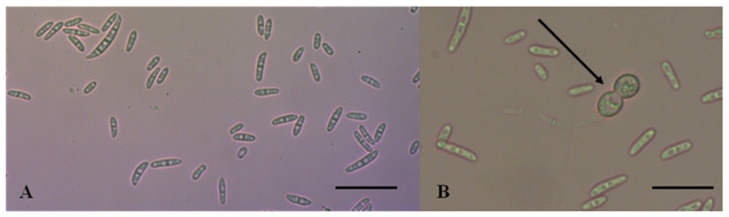
Morphological characteristics of *Fusarium equiseti* isolate obtained in this study. (**A**) micro and macroconidia, (**B**) chlamydospores indicated by the arrow. Scale bars = 10 μm.

Macroconidia were slightly curved, with a tapered and elongated apical cell and prominent foot-shaped basal cell, measuring 18.1–41.6 × 2.5–4.9 μm and showing 5 to 7 septa (Figure 4A). Chlamydospores were abundant, ellipsoidal, or subglobose, formed in clumps or chains and had thick, roughened walls (Figure 4B).

### 3.2. Pathogenicity Tests

**Figure 5:**
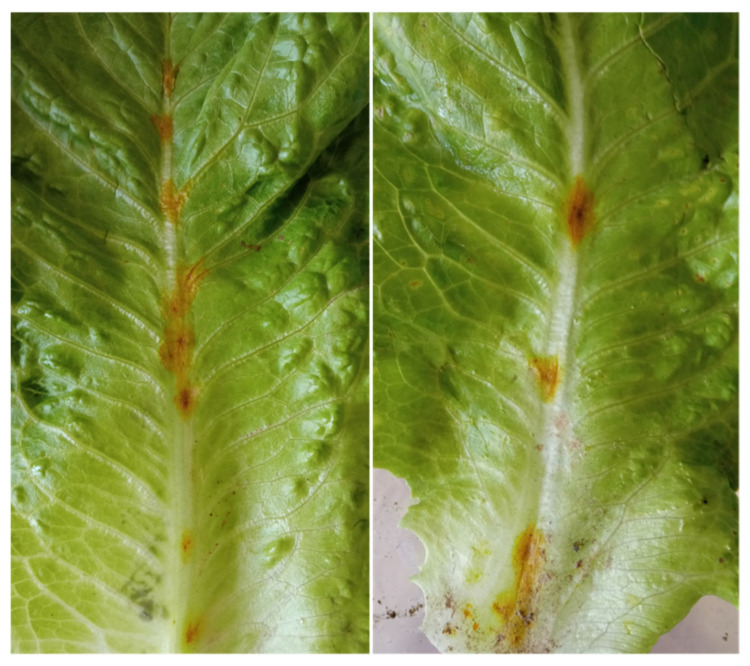
Necrotic spots developed on lettuce leaves ten days after artificial inoculation with *Fusarium equiseti*.

Ten days after inoculation, spots similar to the ones observed in the field developed on all inoculated (with the three isolates) plants, while control plants remained symptomless. More specifically, symptoms recorded were small, brown to black necrotic spots over the foliage. The spots gradually enlarged in size, remaining circular or becoming irregular in shape (Figure 5). FIESC-like isolates were obtained from artificially inoculated plants, while the BLAST search of the GenBank Database, amplifying the *TEF1-α* gene of the obtained isolates, revealed 100% identity with the sequences of various *F. equiseti* isolates. Koch’s postulates were thus fulfilled, and the symptoms observed on lettuce plants in the field were associated with *F. equiseti*.

### 3.3. Molecular Identification and Phylogenetic Analysis

**Figure 6:**
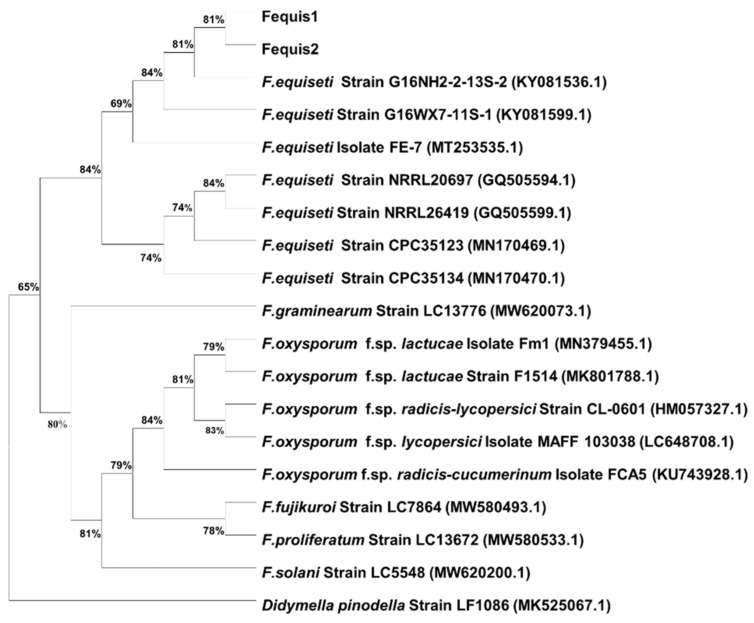
Phylogenetic tree of *Fusarium equiseti* isolates (only representative isolates Fequis1 and Fequis2 are presented) obtained from lettuce plants with foliar disease symptoms constructed using maximum-likelihood analysis, based on the translation elongation factor 1-alpha (*TEF1-a*) gene. Genetic distances were determined according to Kimura’s substitution model and bootstrap support was estimated based on 1000 trials. Numbers in parenthesis are the accession numbers of sequences obtained from the National Center for Biotechnology Information (NCBI). The sequence of *Didymella pinodella* was used as the outgroup for rooting the phylogenetic tree.

Partial amplification of the rDNA ITS1-5.8S-ITS2 region from the 30 isolates obtained in this study yielded an average fragment length of approximately 490 bp. Two representative sequences were deposited in the NCBI database under the accession nos. OP520923-OP520924, and a BLAST search revealed 100% identity with the sequences of various *F. equiseti* isolates, e.g., with that of strain NRRL26419 = CBS307.94 [15]. On the other hand, sequences obtained from the partial amplification of the small subunit (*SSU*) ribosomal RNA (Accession Nos. OP520925-OP520926) exhibited no identity (contained no matching element) when compared with the only three available in NCBI database, *Marssonina panattoniana* sequences (Accession Nos. MK252097, MH866332, and MH854831). In addition, the amplicons of the other three genes used in this study were sequenced [GenBank Accession Nos. OP618093-OP618094 (*TEF1-α*), OP646310-OP646311 (*CAM*), OP680529-OP680530 (*Bt*)] and BLAST search revealed 100% identity with *F. equiseti* sequences (e.g., OL311708 for *TEF1-α*, LN901595 for *CAM* and MT939667 for *Bt*). Partial-sequence alignments of the ITS-rDNA, *CAM*, *Bt*, and *SSU* genes from the *F. equiseti* isolates used in this study (Fequis1 and Fequis2), and the relevant best-hit BLAST *F. equiseti* sequences from the NCBI database are presented in Figure S1. The ML phylogenetic analysis showed that the tested *F. equiseti* isolates clustered together with the sequences of other *F. equiseti* isolates retrieved from the NCBI database, while these isolates separated distinctly from the sequences of other *Fusarium* spp. isolates (Figure 6).

### 3.4. qPCR Primer/Probe Design and Specificity

The qPCR primers and the probe used in this study have been designed to target the translation elongation factor 1-alpha (*TEF1-α*) gene. *TEF1-α* gene is considered as the most informative barcoding marker for the separation of FIESC species [10], as it exhibits higher-sequence polymorphism among species that are closely related [17]. This observation has been recently confirmed in the study of Matić et al. [12] which, among other loci, amplified *TEF1-α* gene for identification of different species of FIESC in leafy vegetables. However, this is the first study in which the *TEF1-α* gene sequences have been used for the design of a primer-probe set to detect *F. equiseti* in a RT-qPCR assay.

Initially, the specificity of the designed primers was tested carrying out a conventional PCR assay. DNA samples obtained from *F. equiseti* and other common soilborne pathogens were included in this step. Conventional PCR provided amplicons of the expected size, and the primers only amplified the respective DNA of *F. equiseti* isolates obtained in this study (Figure S2).

Thereafter, the specificity of the newly developed primer/probe set, targeting *TEF1-α* gene, was confirmed using RT-PCR with the exclusive detection of *F. equiseti* isolates. The assay was specific with the annealing temperature of 55 °C. No amplification product was detected among the other screened soilborne pathogens affecting lettuce cultivation or the other species belonging to the genus *Fusarium*, during the 40 RT-qPCR cycle-assay (Figure S3).

### 3.5. Standard Curve for RT-qPCR and Detection Limit

**Figure 7:**
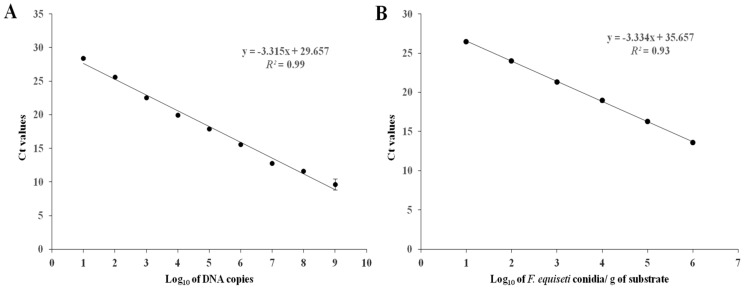
(**A**) Standard curve for *Fusarium equiseti* in a RT-qPCR probe assay. Cycle threshold (Ct) values were plotted against the plasmid DNA serial-dilution concentrations prepared with sterilized water. Each dot represents the mean value of three replicates. Error bars represent the standard deviation. Most error bars were too small to illustrate. (**B**) Validation of the RT-qPCR assay developed in this study. Linear regression of the cycle threshold (Ct) values plotted against the log_10_ of conidia of *Fusarium equiseti*, obtained from the artificially inoculated substrate. The experiment was conducted using three biological replicates for each conidial concentration. Data are means ± standard deviation, although error bars were too small to be visible.

For the development of the quantitative RT-PCR assay, a standard curve was generated by serial ten-fold dilutions (10^1^ to 10^9^ copies/μL) of *in vitro* amplified DNA fragments for *F. equiseti*. The generated standard curve showed a linear dynamic range of amplification over nine orders of magnitude with a slope of −3.315, corresponding to an efficiency of 95.5%, while the linearity of the curve, denoted by the *R*^2^ value, was 0.99 (Figure 7A). Thus, the detection limit of the RT-qPCR assay was 10 copies/μL for *F. equiseti*, indicating that the standard curve is suitable for absolute quantification of the specific pathogen in soil samples.

### 3.6. Validation of the Assay with Artificially Inoculated Substrate

The validation conducted with artificially inoculated substrate showed a negative linear relationship between the log_10_ of the conidia concentration in the substrate and the qPCR cycle threshold for *F. equiseti*. The slope of the linear regression was −3.334, while the *R*^2^ value was 0.93. The amplification efficiency implies that the developed RT-qPCR assay could be used the quantification of the specific pathogen in soil samples. The relationship between the quantification cycle and the logarithm of the concentration of fungal DNA in triplex RT-qPCR settings is presented in Figure 7B.

## 4. Discussion

This is the first comprehensive study aiming to identify the pathogen associated with leaf spot disease symptoms on lettuce in Greece. Our results revealed that the pathogen involved in the observed foliar disease is *F. equiseti*. Until now, in Greece, the specific disease has been attributed to the pathogen *Microdochium panattonianum* (syn. *Marssonina panattoniana*) and has been known as ‘‘lettuce anthracnose’’. However, the results of the amplified product of the small ribosomal subunit (*SSU*) gene of the isolates obtained in this study showed no homology at all with the existing sequences in the NCBI database. In addition, the morphological characteristics and measurements of the isolates tally with those of *F. equiseti* [9,12,15,16].

Nonetheless, the identification based only on morphology traits is considered insufficient, since genetic methods are required to identify the species precisely [9,10,15,16,33]. Hence, in the present study, one nuclear genomic region (ITS-rDNA) and three genes (*TEF1-a*, *CAM*, and *Bt*) were also amplified. A BLAST search of the generated sequences revealed 100% identity with various sequences of *F. equiseti* retrieved from the NCBI database.

In previous studies, multi-locus datasets encompassing the internal transcribed spacer region (ITS), the intergenic spacer region (IGS), and/or several genes such as *TEF1-a*, *CAM*, *Bt*, *LSU*, *RPB1*, and *RPB2* were generated and were very efficient in distinguishing the species belonging to the FIESC [7,12,13,14,15,16]. More specifically, the diversity of FIESC in isolates from lettuce and other leafy vegetables has been presented in the highly informative study of Matić et al. [12]. These authors characterized the isolates obtained from leafy vegetable hosts using, besides morphological characteristics, MLST analyses of four different loci (*TEF1-a*, *CAM*, *Bt*, and IGS).

Although FIESC has recently been the main objective of several research studies [7,12,13,14,15,16], no information is yet available about its potential detection and subsequent quantification in soil samples. For this reason, we attempted to develop a RT-qPCR assay for the detection and quantification of this emerging plant pathogen, using a newly designed primer pair (FeqELf-F and FeqELf-R) and a probe (FeqELf-Pro). The qPCR showed a high level of specificity and sensitivity for the detection of *F. equiseti* in lettuce cultivated fields. The detection limit of the RT-qPCR assay was 10 copies/μL for *F. equiseti*, and the linearity of the curve, denoted by the R squared value (*R*^2^) of the developed standard curve, was 0.99, indicating that this standard curve is reliable for quantification of the target organism in soil samples.

Applied detection methods could be crucial for the reliable identification of the pathogens infecting leafy vegetables, enabling the implementation of preventative management methods [3]. The main method of controlling fungal diseases presented in fields cultivated with leafy vegetables is crop rotation, aiming to keep the inoculum concentration of specific pathogens as low as possible [5]. However, pathogens with a broad range of plant hosts could constitute problematic cases in terms of control. In cases like this, effective monitoring and reliable quantification could contribute to a more effective disease management. For instance, specific and sensitive quantitative PCR assays were developed for the detection and quantification of *R. solani*, causing bottom rot of lettuce, in tissue and soil samples [22]. In this study, qPCR assay was sensitive enough to detect the lowest soil concentration of *R. solani* AG1-IB, capable of inducing symptoms in head lettuce, constituting thus a potential useful tool for the detection and management of lettuce bottom rot disease.

Accurate identification of *Fusarium* species using morphological criteria and/or molecular methods is laborious and at most times ambiguous [9,23]. In addition, as O’Donnell et al. [34] have already pointed out, the top BLAST matches upon querying the GenBank database may be imprecise or even confusing due to originally misidentified sequences and/or updated taxonomy. Therefore, the quantitative PCR has turned into a significant laboratory method for the detection and quantification of specific DNA targets, an assumption that has been confirmed recently for *F. avenaceum* [24]. In that study, a primer pair, designed from the *TEF1-α* gene sequence, has been effectively used for the quantification of *F. avenaceum*, a cosmopolitan pathogen, in soil and seed samples.

Most likely, the recorded emergence of *F. equiseti* on lettuce in Greece could be due to its altered pathogenic status, from sporadic weak to principal plant pathogen, and its extended plant host range [12]. Moreover, the emergence of the specific fungus on new plant hosts could be associated with environmental changes [12]. Increased disease incidence and severity, caused by *F. equiseti*, has already been reported on wild rocket and radish under elevated average temperatures and CO_2_ concentrations [35]. Garibaldi et al. [36] pointed out the effect of temperature and leaf wetness duration on the incidence and severity of leaf spot caused by *F. equiseti* on lettuce and wild rocket under controlled conditions. In their study, lettuce was more susceptible to this pathogen, exhibiting higher disease index and disease severity, at 25–30 °C and after a few hours (1–3 h) of leaf wetness. On the contrary, in case of lower temperatures (15–30 °C), at least 12 h of leaf wetness were needed in order to achieve favorable conditions for disease development and significant losses.

In addition, *F. equiseti* could be transmitted by seeds of several leafy vegetable hosts, such as wild rocket [3]. This epidemiological aspect, along with the climate change scenario, could constitute a potential cause for the outbreak of *F. equiseti* on leafy vegetables [4,12]. Interestingly enough, this broad-range plant pathogen, once introduced in a field, can survive on infested plant debris or in soil, making rotations in severely infected areas of limited success [3,4].

In our opinion, as the identification of FIESC species based only on morphological characteristics is considered problematic [7], and the molecular identification requires the amplification of more than one gene [7,12,13,14,15,16], a sensitive, specific, and reproducible RT-qPCR could provide reliable detection of the specific pathogen. Henceforth, the RT-qPCR method developed in this study could be used for the quantification of *F. equiseti* in soil samples, collected from fields cultivated with leafy vegetables or even with other plant hosts.

Disease diagnosis and plant pathogen detection are key factors to important agronomic decisions, such as selection of plant species for cultivation and selection of the most efficient management method [37]. The broad plant host range of *F. equiseti*, in addition to the environmental conditions that are favorable for the development of the disease, make its control difficult and most times inefficient [5]. As it is impractical to control environmental parameters (mainly temperature and relative humidity [36]), growers and agriculturists usually rely on the chemical management of the pathogen, which is still under consideration [5]. Therefore, the probe-based qPCR assay developed in this manuscript could constitute a potential useful procedure for the detection and quantification of *F. equiseti* in soil samples and afterwards the evaluation of the adopted management methods.

## Figures and Tables

**Table 1:** TaqMan *Fusarium equiseti* primer and probe set used in the RT-qPCR assay.

Locus	Primer/ Probe Name	Sequence (5′ to 3′)	GC%	Annealing Temperature (°C)	Amplicon Length (bp)
Translation elongation factor (*TEF1-α*)	FeqELf-F	GATCCATCATTCGAATCAGTCTCG	45.8		128
	FeqELf-R	AAGCGCGTGTCACTCGAGTA	55.0	55	
	FeqELf-Pro	6-FAM/AATATGCGC/ZEN/CTGTTACCCCGCTCGAGTA/3IABkFQ	53.6		

## Data Availability

The data presented in this study are available in this article.

## Supplementary Materials

The following supporting information can be downloaded at: https://www.mdpi.com/article/10.3390/pathogens11111357/s1, Figure S1: Partial sequence alignments of the (A) ITS-rDNA, and (B) *CAM*, (C) *Bt*, and (D) *SSU* genes from the *F. equiseti* isolates used in this study (Fequis1 and Fequis2) and the relevant best hit BLAST *F. equiseti* sequences from the NCBI database Figure S2: PCR amplicons of 128 bp obtained with the *F. equiseti* specific primers designed in this study. No amplified product was observed for the DNA samples extracted from other soilborne pathogens, except primer dimers in *Fusarium oxysporum* f.sp. *lactucae* lane. Size marker 100 bp. Figure S3: Analysis of *Fusarium equiseti* primers-probe specificity. Amplification was observed only on the targeted *Fusarium equiseti* species (red arrow). No amplification was recorded on off-target soilborne fungal species and no-template samples (yellow arrow) included in the qPCR assay.
