# Supplementary material for: Fusarium equiseti as an Emerging Foliar Pathogen of Lettuce in Greece: Identification and Development of a Real-Time PCR for Quantification of Inoculum in Soil Samples
Source: Pathogens. 2022 Nov 15;11(11):1357. doi: 10.3390/pathogens11111357 (PMC9699145; doi:10.3390/pathogens11111357)
Supplement: Supplementary file 1 [file pathogens-11-01357-s001.zip › Pathogens-Figure S1.pdf]

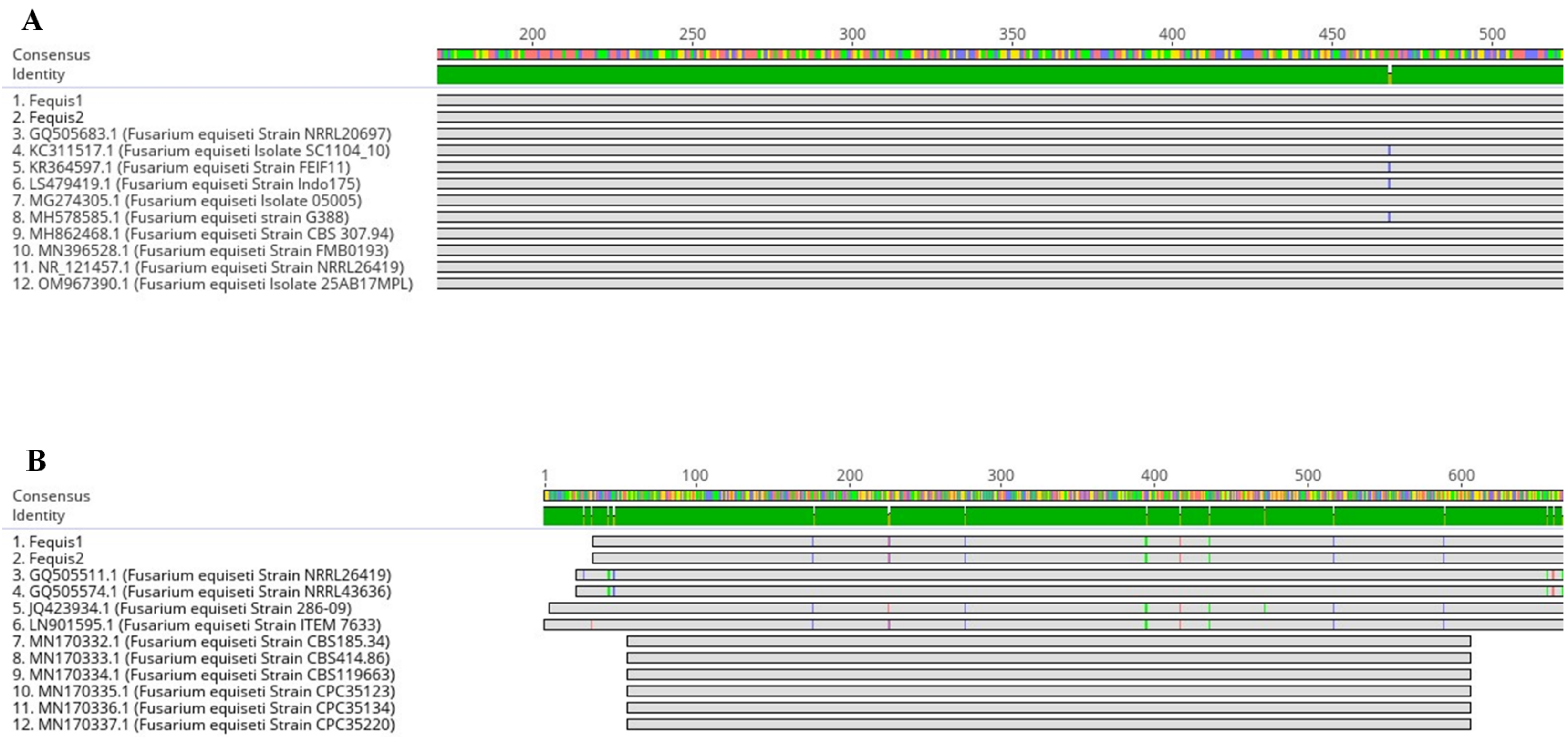
A
200
250
300
350
400
450
500
Consensus
Identity
1. Fequis1
2. Fequis2
3. GQ505683.1 (Fusarium equiseti Strain NRRL20697)
4. KC311517.1 (Fusarium equiseti Isolate SC1104_10)
5. KR364597.1 (Fusarium equiseti Strain FEIF11)
6. LS479419.1 (Fusarium equiseti Strain Indo175)
7. MG274305.1 (Fusarium equiseti Isolate 05005)
8. MH578585.1 (Fusarium equiseti strain G388)
9. MH862468.1 (Fusarium equiseti Strain CBS 307.94)
10. MN396528.1 (Fusarium equiseti Strain FMB0193)
11. NR_121457.1 (Fusarium equiseti Strain NRRL26419)
12. OM967390.1 (Fusarium equiseti Isolate 25AB17MPL)
B
1
100
200
300
400
500
600
Consensus
Identity
1. Fequis1
2. Fequis2
3. GQ505511.1 (Fusarium equiseti Strain NRRL26419)
4. GQ505574.1 (Fusarium equiseti Strain NRRL43636)
5. JQ423934.1 (Fusarium equiseti Strain 286-09)
6. LN901595.1 (Fusarium equiseti Strain ITEM 7633)
7. MN170332.1 (Fusarium equiseti Strain CBS185.34)
8. MN170333.1 (Fusarium equiseti Strain CBS414.86)
9. MN170334.1 (Fusarium equiseti Strain CBS119663)
10. MN170335.1 (Fusarium equiseti Strain CPC35123)
11. MN170336.1 (Fusarium equiseti Strain CPC35134)
12. MN170337.1 (Fusarium equiseti Strain CPC35220)

C

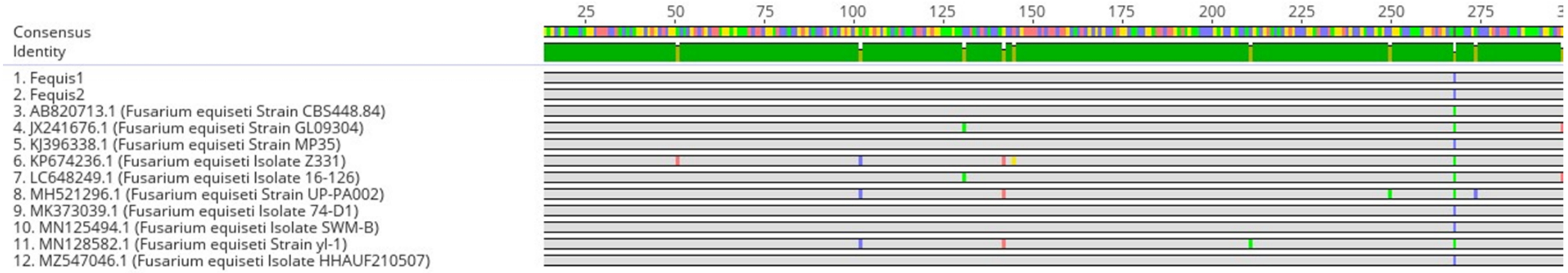

D

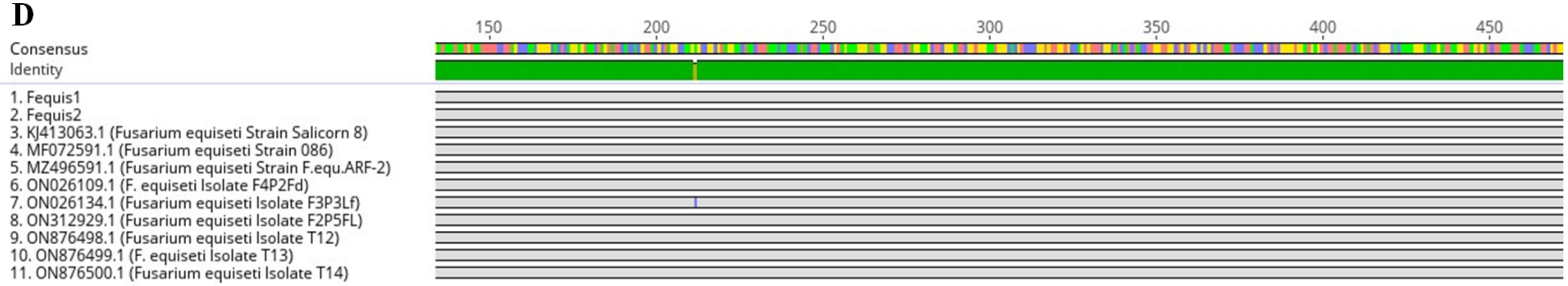

Figure S1. Partial sequence alignments of the A) ITS-rDNA, and B) *CAM*, C) *Bt*, and D) *SSU* genes from the *F. equiseti* isolates used in this study (Fequis1 and Fequis2) and the relevant best hit BLAST *F. equiseti* sequences from the NCBI database.
