# Supplementary material for: Fusarium equiseti as an Emerging Foliar Pathogen of Lettuce in Greece: Identification and Development of a Real-Time PCR for Quantification of Inoculum in Soil Samples
Source: Pathogens. 2022 Nov 15;11(11):1357. doi: 10.3390/pathogens11111357 (PMC9699145; doi:10.3390/pathogens11111357)
Supplement: Supplementary file 1 [file pathogens-11-01357-s001.zip › Pathogens-Figure S2.pdf]

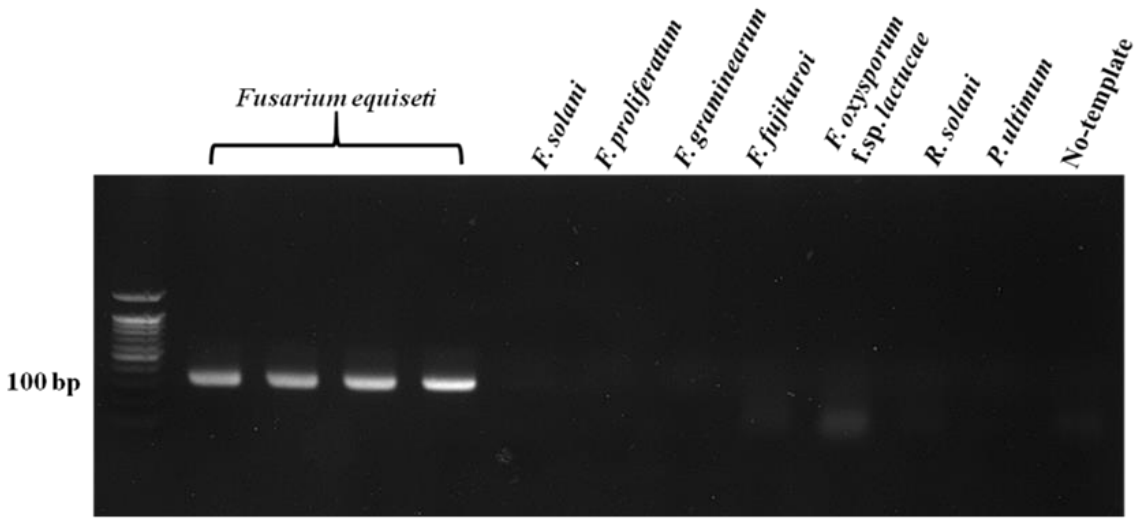

Figure S2. PCR amplicons of 128 bp obtained with the *F. equiseti* specific primers designed in this study. No amplified product was observed for the DNA samples extracted from other soilborne pathogens, except primer dimers in *Fusarium oxysporum* f.sp. *lactucae* lane. Size marker 100 bp.
