# Supplementary material for: Fusarium equiseti as an Emerging Foliar Pathogen of Lettuce in Greece: Identification and Development of a Real-Time PCR for Quantification of Inoculum in Soil Samples
Source: Pathogens. 2022 Nov 15;11(11):1357. doi: 10.3390/pathogens11111357 (PMC9699145; doi:10.3390/pathogens11111357)
Supplement: Supplementary file 1 [file pathogens-11-01357-s001.zip › Pathogens-Figure S3.pdf]

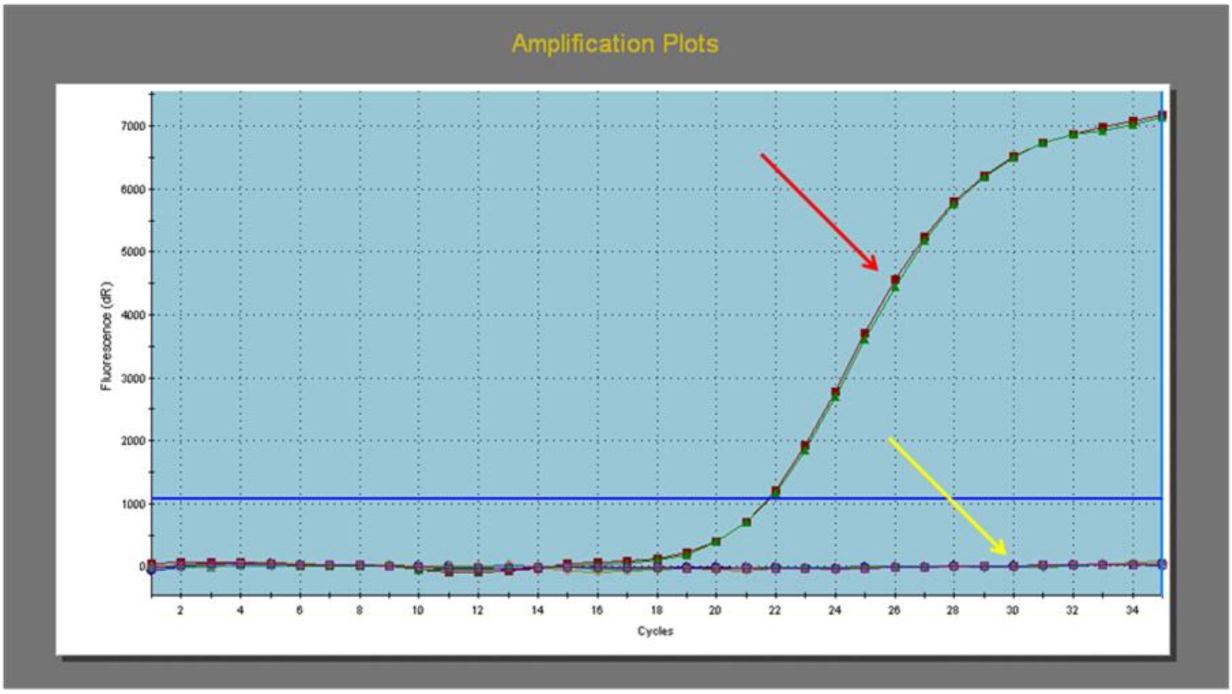

Figure S3. Analysis of *Fusarium equiseti* primers-probe specificity. Amplification was observed only on the targeted *Fusarium equiseti* species (red arrow). No amplification was recorded on off-target soilborne fungal species and no-template samples (yellow arrow) included in the qPCR assay.
